# Supplementary material for: Antibiotic Prescribing Practices for Treating COVID-19 Patients in Bangladesh
Source: Antibiotics (Basel). 2022 Oct 4;11(10):1350. doi: 10.3390/antibiotics11101350 (PMC9598521; doi:10.3390/antibiotics11101350)
Supplement: Supplementary file 1 [file antibiotics-11-01350-s001.zip › antibiotics-1905690-supplementary.pdf]

Title of the Study: Antibiotic prescribing practices for COVID-19 patients in Bangladesh: A survey among physicians

Introduction:

The ongoing COVID-19 pandemic has resulted in around 222 million confirmed cases and more than 4.5 million deaths globally. Most of the people infected with COVID-19 experience mild to moderate respiratory illness and recover without requiring special treatment. However, some infected persons become seriously ill and require medical attention. To ensure a proper clinical treatment for COVID-19 infected patients, different national and international health bodies have recommended clinical guidelines. However, as COVID-19 is a new disease, these protocols are not routinely followed in many countries around the world. Due to absence of an effective treatment, healthcare personnel (HCP) are prescribing varieties of antibiotics for treating COVID-19 patients. There is limited information about physicians' practice around use of antibiotics for COVID-19 patents.

Objective of the study:

We are conducting this online/questionnaire-based survey to understand the trend of prescribing antibiotics among Bangladeshi registered physicians in the treatment of COVID-19 patients in both hospital and home settings.

Methods:

Participation in this survey will take approximately 10 minutes time. We will request you to answer around 28 questions which includes demographic information, antibiotic prescribing status and your suggestion for use of antibiotic for COVID-19 patients.

Why have you been requested to participate in the study?

As you are a practicing clinician based in Bangladesh, we would like to kindly invite you to participate in our study. Your cordial participation and contribution would help us to attain an enriched data that will help to design better communication strategies to promote appropriate use of antimicrobials for COVID-19 patients.

Privacy, anonymity and confidentiality:

We will keep confidentiality of all the information provided by you including any survey data, and all of your responses will be anonymous. Data generated from this study would only be used for the purpose of formulating the results of this research. We will not collect any individual level information that can be linked you.

Right not to participate and withdraw:

Your participation in this survey is completely voluntary. You may choose not to participate or answer any or all of the questions that will be asked about your experience. You can withdraw your participation at any time, even in the middle of the survey. You have the right to refuse participation in this study.

Risks and benefits:

You would not face any potential risk for participating in this survey. You also would not be provided with any monetary incentive or any other direct or indirect facilities for participating in the study.

Contact point for query:

If you have any questions about this study, please contact our Principal Investigator, Utpal Kumar Mondal on email: utpal.sp1980@gmail.com. If you have difficulty in completing the survey, please contact Utpal Kumar Mondal on email: utpal.sp1980@gmail.com or Dr. Tahmidul Haque on email: aunitahmid83@gmail.com

If you agree to participate in our study, please put your specimen signature with date at the bottom of the consent paper.

Thank you for your contribution to this research project.

Yours Sincerely,

On behalf of the Research Team

Dr. Nusrat Homaira, Senior Lecturer, Discipline of Pediatrics, The University of New South Wales (UNSW), and Respiratory Researcher, Sydney Children's Hospital

Md Saiful Islam, University of New South Wales (UNSW)

Dr. Zakiul Hassan, University of Oxford

Utpal Kumar Mondal, Griffith University, Australia

Dr. Tahmidul Haque, BRAC James P Grant School of Public Health

Md. Abdullah Al Jubayer Biswas, University of Dhaka

Signature of the Participant

Signature of the Investigator

Antibiotic prescribing practices for COVID-19 patients in Bangladesh: A survey among physicians

Email address of the participant:

A. Questionnaire Identification

	Questions and Instructions	Responses and Direction
1.	Type of healthcare setting where you work (Multiple answers are accepted)	A. Primary (Subdistrict, Union and Village level)<br>B. Secondary (District Hospitals; General Hospitals)<br>C. Tertiary (Teaching Hospitals; Specialized Hospitals, Medical College Hospitals)<br>D. COVID-19 dedicated hospitals<br>E. Private hospitals
2.	Type of ward where you work	A. Inpatient<br>B. Outpatient<br>C. General ward<br>D. ICU<br>E. COVID-19 ward
3.	Name of the District where you work as a clinical practitioner:	

B. Antibiotic prescribing status on COVID-19 patient in Bangladesh

Antibiotic means all the drugs used in treatment against growth and clinical manifestation of any microorganism. Henceforth, all antimicrobial, antimalarial, antiparasitic and antiprotozoal drugs would be considered as antibiotics in this survey.

1.	Did you treat any COVID-19 patients since the first COVID-19 case was identified in Bangladesh?	1. Yes<br>2. No
2.	COVID-19 is a viral illness.	A. Strongly Agree<br>B. Agree<br>C. Neither Agree or Disagree<br>D. Disagree<br>E. Strongly Disagree
3.	There is currently no effective treatment for COVID-19.	A. Strongly Agree<br>B. Agree<br>C. Neither Agree or Disagree<br>D. Disagree<br>E. Strongly Disagree
4.	Early symptomatic and supportive treatment can help most patients recover from COVID-19.	A. Strongly Agree<br>B. Agree<br>C. Neither Agree or Disagree<br>D. Disagree<br>E. Strongly Disagree

5.	Antibiotics can prevent COVID-19.	A. Strongly Agree<br>B. Agree<br>C. Neither Agree or Disagree<br>D. Disagree<br>E. Strongly Disagree
6.	Antibiotic is recommended for all COVID-19 patients.	A. Strongly Agree<br>B. Agree<br>C. Neither Agree or Disagree<br>D. Disagree<br>E. Strongly Disagree
7.	Persons with COVID-19 cannot transmit the virus to other people when they have been treated with antibiotics.	A. Strongly Agree<br>B. Agree<br>C. Neither Agree or Disagree<br>D. Disagree<br>E. Strongly Disagree

8.	When should you prescribe antibiotics for COVID-19 patients?<br>**(Please provide a response for each of the options below)**	Strongly Agree	Agree	Neither Agree or Disagree	Disagree	Strongly Disagree
	A. As soon as the patient has COVID-19 positive test results					
	B. Patient has comorbidity (such as heart disease, diabetes, asthma, chronic kidney disease, cancer)					
	C. Patient with respiratory problems (such as symptoms of pneumonia, chest tightness, breathing difficulty, wheezing, persistent coughing)					
	D. Elderly patient (>60 years)					
	E. Adult patient (>35-60 years)					
	F. Young patient (18-35 years)					
	G. Children (<18 years)					
	H. Pregnant women					
	I. Patients treated outside of healthcare settings					
	J. Patient with high fever					
	K. Patient with myalgia					
	L. Patient with loose motion					
	M. Patient with anosmia					
	N. Patient with secondary bacterial infection					

9.	How long do you think antibiotic treatment should last for COVID-19 patient? **(Please respond to each of the options below)**	Strongly Agree	Agree	Neither Agree or Disagree	Disagree	Strongly Disagree
	A. 3-5 days					
	B. 5-7 days					
	C. More than 7 days					
	D. When the patient doesn't have a fever anymore					
	E. When patient doesn't have any symptoms					
	F. When patient does not need to be admitted to the hospital					
10.	Antibiotics can reduce the duration of COVID-19 symptoms.	A. Strongly Agree<br>B. Agree<br>C. Neither Agree or Disagree<br>D. Disagree<br>E. Strongly Disagree				
11.	It is necessary to conduct laboratory tests before prescribing antibiotics to COVID-19 patients.	A. Strongly Agree<br>B. Agree<br>C. Neither Agree or Disagree<br>D. Disagree<br>F. Strongly Disagree				
12.	Laboratory test that should be done before administering antibiotics?<br><br>**(Please respond to each of the options below)**	Strongly Agree	Agree	Neither Agree or Disagree	Disagree	Disagree
	A. Complete blood count (CBC)					
	B. Levels of hemoglobulin					
	C. Creatinine					
	D. Bilirubin					
	E. Alanine aminotransferase (ALT)					
	F. C reactive protein					
	G. X-ray of chest					
	H. CT scan of the chest					
	I. Blood culture					
	J. ECG					
	K. Blood culture and sensitivity test (C/S)					

13.	In the absence of laboratory confirmation of bacterial infection, what are the symptoms which makes you prescribe antibiotic for COVID-19 patients?<br><br>**(Multiple answers are accepted)**	1. High fever<br>2. Symptoms of pneumonia<br>3. Breathlessness/breathing difficulty<br>4. Chest tightness<br>5. Persistent coughing<br>6. Anosmia<br>7. Loose motion<br>Myalgia<br>8. Other (please specify if any)<br>__________________________
14.	Antibiotics should not be used for COVID-19 patients without clinical evidence of bacterial infection.	A. Strongly Agree<br>B. Agree<br>C. Neither Agree or Disagree<br>D. Disagree<br>E. Strongly Disagree
15.	Antibiotics are routinely used for treating COVID-19 in Bangladesh.	A. Strongly Agree<br>B. Agree<br>C. Neither Agree or Disagree<br>D. Disagree<br>E. Strongly Disagree
16.	What antibiotics have you used/prescribed for treating COVID-19 patients?<br><br>**(Multiple answers are accepted)**	

	**COVID-19 patients with mild to moderate illness:**<br><br>Clinical definition of mild to moderate illness: In a mild case, clinical symptoms are mild and no evidence of pneumonia is found. Symptoms may be fever, myalgia, sore throat, fatigue, cough, loss of taste or smell, headache, diarrhoea, vomiting, rhinorrhoea, abdominal pain and skin lesions. In a moderate case, adolescent presents with clinical signs of pneumonia (fever, cough, dyspnoea, fast breathing). In case of children, clinical signs of non-severe pneumonia (cough or difficulty in breathing along with fast breathing and/or chest indrawing). Breathing rate >= 60/min in <2 months old, >= 50/min in 2-5 months old and >=40/min in 1-5 years old.	**COVID-19 patients with severe illness:**<br><br>Clinical definition of severe illness: In adults, fever, suspected respiratory infection, respiratory rate > 30/min in any reading, severe respiratory distress (SPO2 <90% on room air). In case of children, clinical signs of pneumonia (cough and difficulty in breathing) + at least one of the followings; 1. central cyanosis or SPO2 <90% on room air, severe respiratory distress (i.e., fast breathing, grunting and very severe chest indrawing) and general danger signs like inability to breastfeed, lethargy or convulsions. 2. Fast breathing (in breaths/min): >= 60/min in <2 months old, >= 50/min in	**COVID-19 patients with critical illness:**<br><br>Clinical definition of critical illness: Respiratory failure and requiring mechanical ventilation, development of ARDS (acute respiratory distress syndrome), sepsis, septic shock and any organ failure that requires ICU care.

		2-5 months old and<br>>=40/min in 1-5 years old.	
	A. Ceftriaxone (cephalosporin<br>B. Meropenem (carbapenem)<br>C. Levofloxacin (fluoroquinolones)<br>D. Moxifloxacin(fluoroquinolones)<br>E. Doxycycline (Tetracyclines)<br>F. Azithromycin (macrolide antibiotics)<br>G. Amoxicillin (Penicillins)<br>H. Hydroxychloroquine (Antimalarials)<br>I. Gentamicin (Aminoglycoside)<br>J. Linezolid (Oxazolidinones)<br>K. Glycopeptides<br>L. Anti protozoal drug (Nitroimidazoles)<br>M. Antiparasitic drug (Ivermectin)<br>N. None of the above	A. Ceftriaxone (cephalosporin<br>B. Meropenem (carbapenem)<br>C. Levofloxacin(fluoroquinolones)<br>D. Moxifloxacin(fluoroquinolones)<br>E. Doxycycline (Tetracyclines)<br>F. Azithromycin (macrolide antibiotics)<br>G. Amoxicillin (Penicillins)<br>H. Hydroxychloroquine (Antimalarials)<br>I. Gentamicin (Aminoglycoside)<br>J. Linezolid (Oxazolidinones)<br>K. Glycopeptides<br>L. Anti protozoal drug (Nitroimidazoles)<br>M. Antiparasitic drug (Ivermectin)<br>N. None of the above	A. Ceftriaxone (cephalosporin<br>B. Meropenem (carbapenem)<br>C. Levofloxacin(fluoroquinolones)<br>D. Moxifloxacin(fluoroquinolones)<br>E. Doxycycline (Tetracyclines)<br>F. Azithromycin (macrolide antibiotics)<br>G. Amoxicillin (Penicillins)<br>H. Hydroxychloroquine (Antimalarials)<br>I. Gentamicin (Aminoglycoside)<br>J. Linezolid (Oxazolidinones)<br>K. Glycopeptides<br>L. Anti protozoal drug (Nitroimidazoles)<br>M. Antiparasitic drug (Ivermectin)<br>N. None of the above
17.	Have you ever prescribed combinations of antibiotics for COVID-19 Patient?	A. Yes<br>B. No<br>C. Don't know	
18.	If yes for question 17, how many antibiotics have you combined for prescribing COVID-19 patients?	1. 2<br>2. 3<br>3. 4<br>4. 5<br>5. 6<br>6. 7<br>7. More than 7	

C. Demographic information of the respondent

	Questions and Instructions	Responses and Direction
1	Gender	1- Male 2. Female 3. Prefer not to disclose
2	Age	_______________ (in year)

3	Highest level of education received:	A. MBBS<br>B. Diploma<br>C. MD<br>D. MS<br>E. MCPS<br>F. FCPS<br>G. MRCP<br>H. FRCP<br>I. MRCS<br>J. FRCS<br>K. Others (please specify if any)<br>_______________________
4	Designation of the physician:	1. Intern Doctor<br>2. HMO (Honorary Medical Officer)<br>3. Post-Graduation Trainee/Resident<br>4. Medical Officer/Assistant Surgeon<br>5. Register<br>6. Consultant<br>7. Assistant Professor<br>8. Associate Professor<br>9. Professor
5	Years of experience as physicians<br>(Years and months)	_______years _______months
